# Monocyte Subset Recruitment Marker Profile Is Inversely Associated With Blood ApoA1 Levels

**DOI:** 10.3389/fimmu.2021.616305

**Published:** 2021-02-26

**Authors:** Vyoma K. Patel, Helen Williams, Stephen C. H. Li, John P. Fletcher, Heather J. Medbury

**Affiliations:** ^1^ Vascular Biology Research Centre, Department of Surgery, Westmead Hospital, Westmead, NSW, Australia; ^2^ The University of Sydney, Westmead Clinical School, Westmead, NSW, Australia; ^3^ Western Sydney University, Blacktown/Mt Druitt Clinical School, Blacktown Hospital, Blacktown, NSW, Australia; ^4^ Institute of Clinical Pathology and Medical Research, Westmead Hospital, Westmead, NSW, Australia

**Keywords:** monocytes, atherosclerosis, dyslipidemia, adhesion, migration, cardiovascular disease

## Abstract

Dyslipidemia promotes development of the atherosclerotic plaques that characterise cardiovascular disease. Plaque progression requires the influx of monocytes into the vessel wall, but whether dyslipidemia is associated with an increased potential of monocytes to extravasate is largely unknown. Here (using flow cytometry) we examined recruitment marker expression on monocytes from generally healthy individuals who differed in lipid profile. Comparisons were made between monocyte subsets, participants and relative to participants’ lipid levels. Monocyte subsets differed significantly in their expression of recruitment markers, with highest expression being on either the classical or non-classical subsets. However, these inter-subset differences were largely overshadowed by considerable inter-participant differences with some participants having higher levels of recruitment markers on all three monocyte subsets. Furthermore, when the expression of one recruitment marker was high, so too was that of most of the other markers, with substantial correlations evident between the markers. The inter-participant differences were explained by lipid levels. Most notably, there was a significant inverse correlation for most markers with ApoA1 levels. Our results indicate that dyslipidemia, in particular low levels of ApoA1, is associated with an increased potential of all monocyte subsets to extravasate, and to do so using a wider repertoire of recruitment markers than currently appreciated.

## Introduction

Monocyte recruitment into the blood vessel wall is an essential step for atherosclerotic plaque development which characterizes cardiovascular disease (CVD). Monocyte transformation into macrophages, particularly adoption of an inflammatory phenotype, promotes plaque progression including acquisition of an unstable plaque morphology which can lead to clinical events (1, 2). In murine models of atherosclerosis, monocyte accumulation is proportional to lesion size (3) and blocking monocyte recruitment reduces plaque progression (4). Similarly, inhibiting monocyte recruitment is considered an attractive target for reducing human plaque development, inflammation and consequently, clinical events (5).

Monocyte recruitment is primarily mediated by two key steps: adhesion and migration (6, 7). Importantly, monocyte firm adhesion is mediated through a well-orchestrated arrangement of various adhesion molecules such as selectins and integrins (6–9) and their transmigration mediated by the chemokine receptors and their associated ligands (10–12). Which specific molecules monocytes use likely varies as they are a heterogenous population with three major subsets identified: Classical (CD14^++^CD16^-^), intermediate (CD14^++^CD16^+^), non-classical (CD14^+^, CD16^++^) (13). The expression of recruitment markers on human monocyte subsets differs (14, 15). In particular, classical monocytes express higher levels of adhesion molecules such as CD62L and chemokine receptors CCR2, CXCR2, whereas CD16+ (intermediate and non-classical) monocytes express higher levels of CCR5 and CX3CR1 (12, 14, 16–18). As such, classical monocytes are considered to have a greater potential to migrate into injured or inflamed tissue than the intermediate and non-classical subsets (19). Indeed, they do so in murine models of inflammation, including models of atherosclerosis (20); accordingly, a lack of classical, but not non-classical monocytes greatly reduce lesion size (21).

Within the circulation itself, monocyte numbers are increased in cardiovascular disease, with an increased proportion of intermediate monocytes associated with occurrence of major cardiovascular events (22, 23) and increased mortality (22, 24). Whether the distinct recruitment marker profile of monocyte subsets is also altered is unclear, but needs to be assessed if monocyte migration, particularly of a specific subset, is to be considered as a therapeutic target for reducing plaque progression.

The increased monocyte count in CVD is related to lipid levels (dyslipidemia), primarily, to low levels of HDL-C that promote monocytosis (25). HDL, through its major protein component apolipoprotein A1 (ApoA1), is also known to have anti-inflammatory properties (26–28), and in line with this, we previously found monocyte inflammatory status, such as production of IL-1β, is related to HDL-C and ApoA1 levels—even for generally healthy individuals (29). Whether dyslipidemia is also accompanied by increased potential for migration into the plaque is important to discern because with an estimated 2 in 3 adults having dyslipidemia (30), influx of inflammatory monocytes into the vessel wall may be silently promoting atherosclerotic plaque development in countless dyslipidemic individuals who are considered otherwise generally healthy. In this study, we assessed monocyte recruitment marker expression in individuals who were generally healthy but differed in lipid levels. The results were compared between monocyte subsets, between participants, and relative to participants’ lipid levels.

## Materials and Methods

### Study Population

This study was approved by the Western Sydney Local Health District (WSLHD) Human Research Ethics Committee. Informed signed consent was obtained from all participants. We recruited individuals (n = 30) who were in generally good health but differed in lipid levels – with a wide range of lipid levels achieved by including participants visiting the Westmead Lipid clinic. Exclusion criteria included: a documented history of CVD, diagnosed hypertension, diabetes mellitus (Type I or II), a current acute or chronic inflammatory disease (C-reactive protein (CRP) > 5.0 mg/L), being a current smoker, and/or taking lipid-lowering or anti-inflammatory medication.

### Biochemical and Lipid Measurements

Peripheral blood samples were collected from overnight-fasted participants. Leukocyte counts, total cholesterol (TC), high-density lipoprotein cholesterol (HDL-C), low-density lipoprotein cholesterol (LDL-C), triglyceride (TG), apolipoprotein A1 (ApoA1), apolipoprotein B (ApoB), glucose and CRP were measured using standard laboratory methods at the Institute for Clinical Pathology and Medical Research (ICPMR) at Westmead Hospital.

### Assessment of Expression of Surface Adhesion Molecules and Chemokine Receptors on Monocyte Subsets

Surface marker assessment was performed by whole blood flow cytometry on K2EDTA-anti-coagulated blood. Whole blood aliquots (50 µL) were stained with anti-CD14-V450 (BD Pharmingen), anti-CD16-APC (Abcam) and anti-HLA-DR Per-CP (Biolegend) to identify monocyte subsets. PE-conjugated antibodies against surface adhesion molecules, selectins: CD44 and CD62L; integrins: CD11a, CD11b, CD11c, CD18, CD29, CD49d, and chemokine receptors: CD182, CD183, CD184, CD195, CD197, CCR2, CX3CR1 (all PE antibodies from BD Pharmingen) were also added. The tubes were then incubated for 30 min at 4°C in the dark. PE-conjugated matching isotype controls were used to determine relative degree of surface marker expression. The cells were fixed, and red blood cells lysed, by the addition of 250 µL Optilyse C (Beckman Coulter).

### Flow Cytometry

Flow cytometry was used to detect monocyte surface marker expression. Data was collected on a BD FACS™ Canto II flow cytometer (BD) using FACSdiva software (v6.0, BD). At least 5,000 events—based on cells falling in a strong CD14 positive gate on SSC-A vs. CD14 density plot—were recorded.

CompBeads (BD), were used to generate a compensation matrix which was applied before data analysis, which was performed using FlowJo^®^ software (v10.1r5, Tree Star, USA). The gating strategy for identifying the monocytes subsets, while excluding potential contaminating cell types including B cells, T cells, neutrophils and NK cells, was as previous (29). The relative level of expression was determined by the ratio of the geometric mean fluorescence intensity (MFI) of the marker of interest over the MFI of the isotype control as previously reported (31).

### Statistical Analysis

SPSS software (v25, IBM Corporation) was used for statistical analysis. Data for adhesion molecule and chemokine receptor expression are shown as mean ± SD unless otherwise stated. The data were log transformed to stabilise the variance prior to analysis. Comparisons between monocyte subsets were performed using ANOVA followed by *post-hoc* Tukey’s test. The differences between the monocyte subsets were back transformed to provide fold changes and associated 95% confidence intervals (CIs). Comparisons between sexes were conducted using the Student’s t-test for normally distributed data. Associations between monocyte subsets and monocyte subset adhesion and migration profile and participants’ lipid levels were assessed using Spearman’s rank correlations. All tests were two-tailed and a *p* value of <*0.05* was considered statistically significant.

## Results

### Monocyte Subsets Differentially Express Surface Adhesion Molecules and Chemokine Receptors

Characteristics of the participants are summarized in **Table 1**.

**Table 1:** Characteristics of study participants.

Characteristics of study participants	*n = 30*
**Age** (years)	45 ± 12 (27–68)
**Sex**	
Male	16 (53)
Female	14 (47)
**Blood pressure**	
SBP (mmHg)	121 ± 17 (100–160)
DBP (mmHg)	77 ± 8.6 (64–95)
**Lipid profile**	
TC (mmol/L)	5.3 ± 1.5 (3.6–9.8)
HDL-C (mmol/L)	1.4 ± 0.48 (0.59–2.7)
LDL-C (mmol/L)	3.2 ± 1.4 (1.5–7.9)
Cholesterol/HDL-C Ratio	4.3 ± 2.2 (2.0–11)
Apo A1 (g/L)	1.4 ± 0.45 (0.53–2.5)
Apo B (g/L)	0.92 ± 0.38 (0.29–2.0)
Triglycerides (mmol/L)	1.2 ± 0.73 (0.27–3.6)
Glucose (mmol/L)	4.9 ± 0.87 (3.0–7.1)
**Risk Factors**	
Dyslipidemia	
TC (>5.5 mmol/L)	11 (36)
LDL-C (>3.5 mmol/L)	9 (30)
TG (>2 mmol/L)	3 (10)
HTN (SBP/DBP, mm Hg)	1 (3)
Glucose (> 5.4 mmol/L)	5 (16)

SBP, systolic blood pressure; DPB, diastolic blood pressure; TC, total cholesterol; HDL-C, high-density lipoprotein cholesterol; LDL-C, low-density lipoprotein cholesterol; TG, triglycerides; HTN, hypertension and Apo, Apolipoprotein. Data for age, blood pressure and lipid profile are presented as mean ± SD and (range). Data for sex and risk factors are number of patients and percentage n (%).

**Figure 1:**
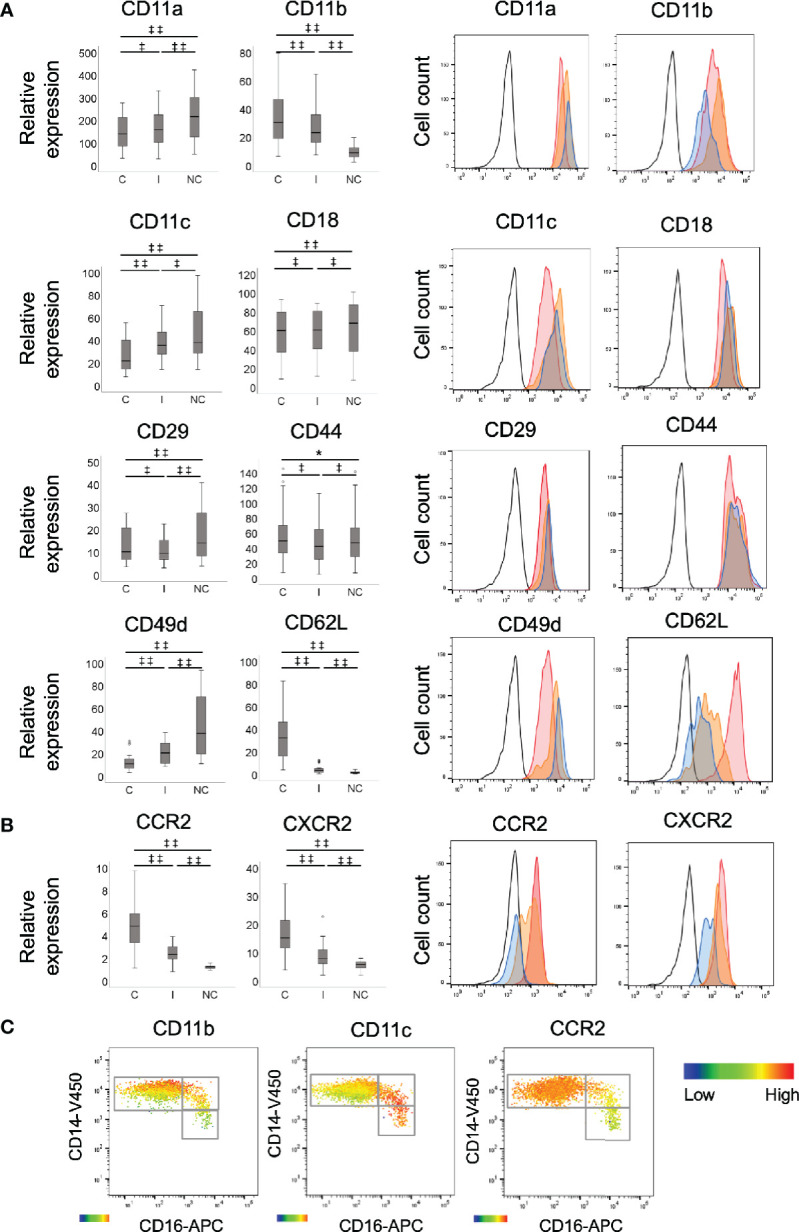
Comparison of recruitment marker expression between monocyte subsets. Expression of **(A)** adhesion molecules, CD11a, CD11b, CD11c, CD18, CD29 (all integrins), CD44 (selectin), CD49d (integrin) and CD62L (selectin) and **(B)** chemokine receptors, CCR2 and CXCR2 by monocyte subsets, measured by flow cytometry. C: classical; I: intermediate; and NC: non-classical, n=30. Data presented as box and whisker plots, with outliers denoted by circles and representative histograms. Black lined histograms: isotype control, red histograms: classical monocytes, orange histograms: intermediate monocytes and blue histograms: non-classical monocytes. The data were log transformed to appropriate normality and in order to stabilise the variance prior to analysis. Statistical calculations of significance were performed using repeated measures ANOVA for significant differences in the relative expression levels of the markers between any 2 monocyte subsets within subject, ^‡‡^p < 0.001; ^‡^p < 0.01; *p < 0.05. The differences between subsets were back transformed to provide fold changes (relative to the isotype control) and associated 95% confidence intervals (CIs). **(C)** Differential expression of recruitment markers on monocyte subsets. Heat map showing the degree of expression from high (red) to low (blue) of integrins, CD11b and CD11c and chemokine receptor, CCR2 on whole blood monocyte population based on CD16 and CD14 expression. The heat maps represent classical, intermediate and non-classical monocytes, respectively. The heat maps were created using Flow Jo software.

The expression of CD11b, CD62L, CCR2, and CXCR2 (CD182) was significantly higher on classical monocytes when compared to intermediate and non-classical monocytes, and higher on the intermediate compared to non-classical (**Figures 1A, B**: all *p<0.001*). Of note, there was no appreciable expression of CCR2 on the non-classical subset and low levels on the intermediate subset for most participants. The expression of CD11a, CD11c, and CD49d was highest on non-classical monocytes followed by the intermediate subset with significant differences between all the subsets (**Figure 1A**: for CD11a: NC/C and NC/I both *p<0.001*, I/C *p<0.01*; CD11c: NC/C and I/C: both *p<0.001* and NC/I: *p<0.01*; CD49d: all *p<0.001*). The expression of CD18 was signficiantly higher on the intermediate and non-classical subsets compared to the classicals, with minimal difference between the intermediate and non-classicals (**Figure 1A**: I/C and NC/C: both *p<0.01)*. The expression of CD29 was highest on non-classicals followed by classicals and then the intermediates (**Figure 1A**: NC/C and NC/I both *p<0.001* and C/I *p<0.01*). All monocyte subsets expressed CD44 with minimal, but still significant, differences in expression between them (**Figure 1A**: C/I and NC/I *p<0.01* and NC/C *p<0.05*). As a whole, no appreciable expression of CCR5 (CD195), CCR7 (CD197), CXCR3 (CD183), CXCR4 (CD184), or CX3CR1 was detected (data not shown) except for CCR5 for which low or minimal levels were detected on the intermediate and non-classical monocyte subsets of 8 individuals. As we had previously found that the expression of monocyte inflammatory markers is not distinct between the subsets, but rather a continuum from the classical, through the intermediate to the non-classical subset (29), we assessed whether the differences in expression of adhesion molecules or chemokine receptors seen between the monocyte subsets were distinct or occurred in a gradual manner. We viewed the expression of adhesion molecules (integrins: CD11b and CD11c) as well as the chemokine receptor, CCR2 within the subsets by heat map on the CD14/CD16 dot plot (**Figure 1C**). The change in expression of CD11b and CD11c between the subsets was independent of their subset divisions as evident by both low and high expression within the classical subset (**Figure 1C**). Interestingly, for CD11b its expression was relative to that of CD14. CCR2 expression followed the traditional classical to non-classical maturation understanding, decreasing through the intermediate to non-classical population (**Figure 1C**).

### The Expression of Recruitment Markers Varies Greatly Between Participants

**Figure 2:**
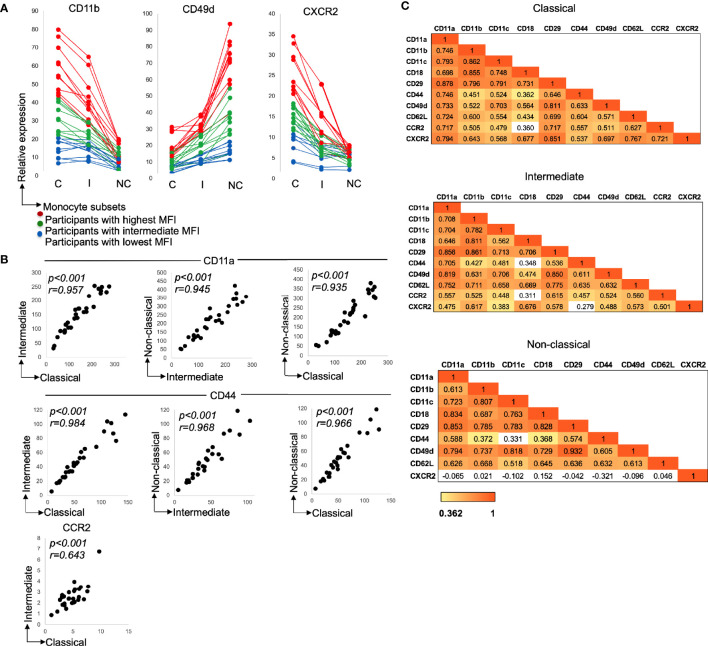
**(A)** Variation in adhesion marker expression between the participants and monocyte subsets. Monocyte subset adhesion molecules, CD11b, CD49d (integrins) and chemokine receptor, CXCR2 expression. Each line on the graph is representative of relative adhesion marker expression from one participant. Participants with the highest marker expression were ranked based on monocyte subsets expressing the highest, CD11b, CD49d, and CXCR2. Red lines show participants with highest CD11b, CD49d, and CXCR2 expression (n = 10), green lines show participants with intermediate CD11b, CD49d, and CXCR2 expression (n=10) and blue lines show participants with lowest geometic mean fluoroscence intensity (MFI), CD11b, CD49d, and CXCR2 expression (n=10). C: Classical; I: intermediate and NC: non-classical. **(B)** Relationship between adhesion molecule expression by the monocyte subsets. Expression of CD11a (all *p < 0.001*; n=30), CD44 (all *p < 0.001*; n=30) and CCR2 (C- *p < 0.001*; n = 30). Data presented as scatter plots. The statistical significance of correlation was determined by Spearman’s rank correlation. All tests were two tailed. r represents correlation coefficient and p represents the statistical significant value. **(C)** Heatmap with correlation matrix. Panel 1: Classical monocytes, 2: Intermediate monocytes and 3: Non-classical monocytes. For all heatmaps, values are Spearman’s rank correlation coefficients. The color in the heatmap represents the degree of correlation. Red (value of 1) represents a stronger correlation coefficient and more significant p value. Yellow (value of 0.362) represents a moderate correlation coefficient and p value of *0.05* or less. White boxes represent non-significant correlations.

There were considerable differences in the expression of recruitment markers between individuals (**Figure 2A**) with marker levels being higher—on all three subsets—for some participants compared to others.

With the increased recruitment marker levels occurring for all subsets, not just one, we looked to see if there was any relationship in the degree of marker expression between the subsets. This was indeed the case, the level of expression of recruitment markers for one subset correlated significantly with that of the next subset (**Figure 2B**: CD11a and CD44, all monocyte subsets—*p<0.001* and CCR2, classical vs. intermediate—*p<0.001*). Note, CCR2 correlated only between the classical and intermediate subsets due to lack of appreciable expression on the non-classical subset.

With the expression of the most recruitment markers varying between participants, the question arises whether an increased level of one marker is associated with increased levels of others. This was assessed (for each subset), and indeed, numerous significant moderate and strong correlations were observed (**Figure 2C**). Correlations were evident not just between the adhesion molecules (e.g. CD11a and all other adhesion markers for each subset) or between the chemokine receptors (CCR2 and CXCR2 for the classical and intermediate subsets), but also between adhesion molecules and chemokine receptors (for example CD11a and CXCR2 for the classical and intermediate subsets).

### Altered Lipid Levels May be One Factor Promoting Monocyte Subset Recruitment

We then assessed whether the variation seen between the participants was explained by their lipid profile.

**Figure 3:**
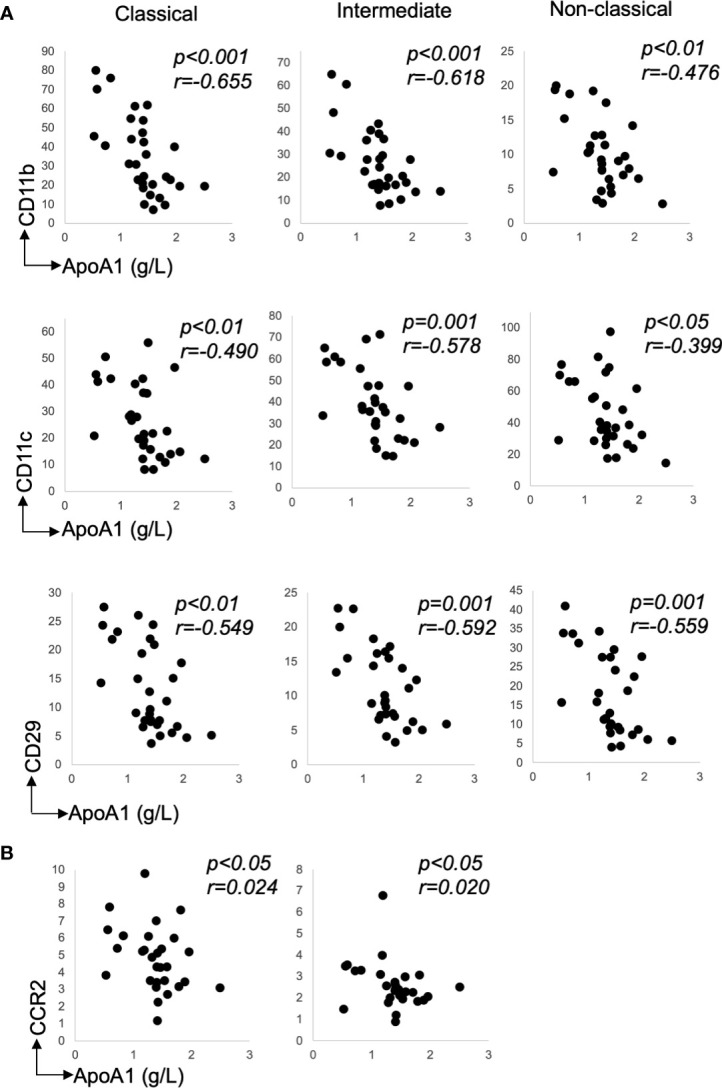
Recruitment marker expression relative to lipid levels. **(A)** Classical, Intermediate and Non-classical correlations for chemokine receptor, CD11b, CD11c, and CD29 with ApoA1, n=30 **(B)** Classical and Intermediate correlations for chemokine receptor, CCR2 with ApoA1, n=30. Data presented as scatter plots. The statistical significance of correlation was determined by Spearman’s rank correlation. All tests were two-tailed. r represents correlation coefficient; p represents level of significance and N.S. represents non-significant.

There were many correlations between the recruitment markers and ApoA1 levels—**Table 2**. Notably, for integrins, CD11b, CD11c, and CD29 expression inversely correlated with ApoA1 levels on all monocyte subsets (**Figures 3A, B**: CD11b: classical and intermediate, *p<0.001* and non-classical, *p<0.01*; CD11c: classical, *p<0.01*, intermediate, *p=0.001* and non-classical, *p<0.05*; and CD29: classical, *p<0.01*, intermediate and non-classical, *p=0.001*). Interestingly, of the integrins, CD29 inversely correlated with glucose levels on all monocyte subsets (**Table 2**, all *p<0.05*).

**Table 2:** Recruitment marker expression relative to lipid levels for each monocyte subset.

		TC (mM)		LDL-C(mM)		HDL-C(mM)		TC: HDL-C ratio		TG(mM)		Apo A1(g/L)		Apo B(g/L)		Apo A1: ApoB ratio		Glucose (mmol/L)	
		*r*	*p*	*r*	*p*	*r*	*p*	*r*	*p*	*r*	*p*	*r*	*p*	*r*	*p*	*r*	*p*	*r*	*p*
**C_CD44**	***n=30***	0.045	0.811	0.080	0.674	−0.080	0.673	0.152	0.424	−0.266	0.156	−0.353	0.056	−0.092	0.630	−0.101	0.595	0.011	0.955
**I_CD44**		0.110	0.564	0.137	0.472	−0.115	0.546	0.197	0.296	−0.208	0.271	−0.361*	0.050	−0.044	0.819	−0.137	0.469	0.101	0.594
**NC_CD44**		0.104	0.583	0.112	0.555	−0.090	0.636	0.190	0.314	−0.219	0.245	−0.334	0.072	−0.080	0.672	−0.940	0.623	0.136	0.473
**C_CD62L**		0.028	0.883	0.190	0.314	−0.104	0.585	0.164	0.388	−0.104	0.583	−0.469**	0.009	−0.121	0.524	−0.208	0.269	−0.292	0.118
**I_CD62L**		0.227	0.228	0.256	0.172	0.029	0.878	0.156	0.411	−0.144	0.449	−0.362*	0.050	−0.023	0.902	−0.189	0.318	−0.150	0.429
**NC_CD62L**		0.234	0.214	0.248	0.186	−0.121	0.524	0.287	0.125	−0.248	0.186	−0.409*	0.025	0.010	0.956	−0.290	0.120	−0.140	0.461
**C_CD11a**		0.080	0.673	0.216	0.251	0.078	0.681	0.059	0.757	−0.238	0.205	−0.444*	0.014	−0.232	0.217	−0.050	0.793	−0.211	0.264
**I_CD11a**		0.116	0.541	0.290	0.120	0.031	0.869	0.130	0.493	−0.184	0.332	−0.414*	0.023	−0.157	0.408	−0.126	0.507	−0.233	0.216
**NC_CD11a**		0.107	0.574	0.307	0.099	0.138	0.468	0.065	0.732	−0.139	0.464	−0.345	0.062	−0.187	0.323	−0.050	0.795	−0.334	0.071
**C_CD11b**		0.224	0.235	0.339	0.067	−0.251	0.182	0.352	0.056	−0.002	0.993	−0.655**	0.000	−0.028	0.885	−0.415*	0.022	−0.321	0.084
**I_CD11b**		0.118	0.536	0.341	0.065	−0.200	0.290	0.276	0.140	−0.002	0.992	−0.618**	0.000	−0.093	0.623	−0.345	0.062	−0.307	0.098
**NC_CD11b**		0.263	0.160	0.279	0.136	−0.047	0.804	0.251	0.180	−0.208	0.270	−0.476**	0.008	0.027	0.889	−0.294	0.115	−0.335	0.070
**C_CD11c**		0.199	0.293	0.285	0.127	−0.105	0.580	0.260	0.166	−0.206	0.275	−0.490**	0.006	0.033	0.864	−0.297	0.112	−0.217	0.249
**I_CD11c**		0.147	0.438	0.203	0.283	−0.186	0.324	0.291	0.119	−0.269	0.151	−0.578**	0.001	−0.006	0.977	−0.325	0.080	−0.290	0.120
**NC_CD11c**		0.200	0.289	0.295	0.114	−0.049	0.795	0.237	0.207	−0.133	0.483	−0.399*	0.029	0.072	0.707	−0.275	0.141	−0.329	0.076
**C_CD18**		0.135	0.477	0.322	0.083	−0.032	0.868	0.139	0.463	0.035	0.853	−0.355	0.054	−0.070	0.713	−0.204	0.281	−0.206	0.275
**I_CD18**		0.186	0.326	0.410*	0.025	0.003	0.988	0.187	0.322	0.107	0.574	−0.234	0.213	0.065	0.733	−0.261	0.163	−0.298	0.109
**NC_CD18**		0.196	0.298	0.455*	0.012	−0.012	0.950	0.198	0.293	0.035	0.855	−0.433*	0.017	−0.097	0.608	−0.246	0.190	−0.340	0.066
**C_CD29**		0.010	0.958	0.267	0.154	−0.010	0.958	0.131	0.491	−0.100	0.599	−0.549**	0.002	−0.217	0.249	−0.168	0.376	−0.375*	0.041
**I_CD29**		0.108	0.571	0.348	0.060	−0.056	0.768	0.205	0.277	−0.067	0.723	−0.592**	0.001	−0.171	0.365	−0.254	0.176	−0.401*	0.028
**NC_CD29**		0.051	0.787	0.215	0.255	0.001	0.997	0.148	0.435	−0.151	0.426	−0.559**	0.001	−0.193	0.308	−0.164	0.387	−0.389*	0.034
**C_CD49d**		0.040	0.834	0.207	0.272	0.077	0.684	0.078	0.682	−0.236	0.208	−0.267	0.153	−0.061	0.748	−0.115	0.547	−0.142	0.454
**I_CD49d**		0.017	0.928	0.197	0.296	−0.008	0.966	0.122	0.522	−0.204	0.278	−0.518**	0.003	−0.168	0.374	−0.156	0.412	−0.331	0.074
**NC_CD49d**		0.022	0.908	0.156	0.410	0.010	0.956	0.115	0.545	−0.250	0.183	−0.479**	0.007	−0.135	0.476	−0.143	0.451	−0.300	0.108
**C_CXCR2**		−0.023	0.902	0.246	0.190	0.063	0.741	0.020	0.915	−0.023	0.904	−0.430*	0.018	−0.225	0.231	−0.055	0.773	−0.320	0.085
**I_CXCR2**		0.002	0.991	0.190	0.314	0.219	0.245	−0.115	0.545	−0.038	0.842	−0.201	0.286	−0.150	0.428	0.061	0.748	−0.226	0.229
**NC_CXCR2**		−0.159	0.401	−0.016	0.934	0.187	0.323	−0.276	0.140	−0.085	0.654	0.040	0.834	−0.224	0.233	0.233	0.215	0.063	0.740
**C_CCR2**		0.158	0.412	0.371*	0.048	0.060	0.757	0.141	0.464	−0.107	0.580	−0.418*	0.024	−0.070	0.719	−0.138	0.476	−0.437*	0.018
**I_CCR2**		0.070	0.719	0.304	0.109	0.056	0.774	0.011	0.953	−0.043	0.826	−0.429*	0.020	−0.097	0.616	−0.043	0.823	−0.163	0.399

C, classical; I, intermediate; NC, non-classical; r, correlation coefficient; p, level of significance (2-tailed), **p ≤ 0.01; *p ≤ 0.05 (Spearman’s rank correlations). Shading indicates the significant values shown in bold. Dark blue indicates the values significant at p ≤ 0.01 and light blue, at p ≤ 0.05.

Correlations between chemokine receptors and lipid levels were evident for e.g. CCR2 and CXCR2—**Table 2**. Notably, the expression of CCR2 positively correlated with the LDL-C levels on classical monocytes only (**Table 2**: *p<0.05*) and inversely correlated with the ApoA1 levels on classical and intermediate subsets (**Table 2**: CCR2 - both *p<0.05*). CXCR2 expression inversely correlated with the ApoA1 levels but only on classical monocytes (**Table 2**: CXCR2—classical: *p<0.05*). The expression of chemokine markers, CCR2 (on the classicals) also inversely correlated with the glucose levels (**Table 2**—*p<0.05*). Note, while there was a spread in participants’ ages—**Table 1**, there was no association between age or sex and monocyte recruitment marker profile (data not shown).

## Discussion

It is well recognized that classical and non-classical monocytes have distinct migratory patterns, and this is attributed to their specific expression of adhesion molecules and chemokine receptors. While significant differences in recruitment marker expression by the monocyte subsets were seen here, they were overshadowed by the considerable differences between participants which suggests that monocyte subset extravasation potential is specific for individuals. The relatively higher expression of recruitment markers on all monocytes of those with low ApoA1 would increase the cells capacity to extravasate and, given the wide range of markers this occurred for, suggests that the subsets could potentially do so using a wider repertoire of recruitment markers than currently appreciated.

The differences in monocyte subset recruitment marker expression is consistent with the understanding that monocyte subsets employ both common and distinct mechanisms in their recruitment to sites of injury (32), with classical monocytes expressing higher levels of CD11b, CD62L, and CCR2 and non-classicals expressing higher levels of CD11a, CD11c, and CD49d. Consistent with other studies (16, 33), the intermediate subset expressed adhesion molecules such as CD11a, CD49d, and CD62L, as well as chemokine receptors, such as CCR2, at a level between that of the classical and non-classical subsets—which reflects their position as an intermediatory cell between the other two populations (34). The differences between subsets were substantial for markers such as CD62L, CD49d, and CCR2, and indeed, for this reason they are often used to define the subsets (14, 18, 35). Though significant differences between the subsets were seen for several other markers, such as CD44 and CD18, the actual differences were quite minimal, and not likely to be of biological significance. The subset differences reflect incremental changes as the cells mature from one subset to the next. Notably however, the subset expression of some makers did not align with the typical classical to non-classical transition pattern. A good example of this being CD11b, where its expression (as seen on a flow cytometry heat-map) was relative to that of CD14. This highlights the considerable heterogeneity within each subset and is consistent with the more recent understanding that there are actually a multitude of monocyte phenotypes (36), or perhaps there is more than one maturation pathway.

Interestingly, we could not detect clear expression of CX3CR1, CCR5, CXCR2, or CXCR4 on the subsets. This seems to contrast with some other studies (14, 16, 18, 37). Antibody-specific differences (such as clone and concentration) are likely to underlie the minimal expression of CX3CR1, which has shown clear expression in other studies (14, 16, 18). Using isolated PBMCs, both CXCR4 and CCR5 have been detected, with CXCR4 expressed by more than 80% of monocytes and CCR5 expressed by approximately 10–40% of monocytes; however, the relative level of expression was not reported in these studies (16). As isolation can lead to an incidental activation of monocytes (38), then the minimal manipulation with our experimental approach, not just choice of antibody, may explain why marker expression here did not mirror findings in isolated monocytes.

Overshadowing the differences between the subsets, was the large variation in recruitment marker expression between participants. Using 30 participants enabled us to see that although there was a consistent trend in the change in marker expression from one subset to the next, there were participants for whom all three of their monocyte subsets expressed a marker at higher levels than all three monocyte subsets of other participants. Thus though monocyte subsets are recognized to have distinct migratory potential (12), their extravasation potential is specific for individuals. Furthermore, the finding that recruitment marker expression on the classicals correlated with that of the intermediates, which then correlated with that of the non-classicals indicates that the migratory potential of the intermediates and non-classicals is determined by that of the classicals. We suggest that the migratory potential of the monocytes is likely to be pre-primed, beforehand, in the bone marrow as several studies show that alterations in the bone marrow are recapitulated as cells differentiate into the monocyte subsets (39–43). Of note, this potential is likely to vary with the presence of co-morbidities or in different disease states. For example, monocyte subset expression of CCR2 and CX3CR1 is altered in infection (44).

The inter-participant differences occuring for most markers raised the question as to whether there were correlations between the expression of the recruitment markers themselves. The considerable number, and strength, of the correlations seen would provide a synergistic increase in migration potential and the fact that the correlations were evident for all three monocyte subsets indicates that the monocyte subsets likely migrate using a wider repertoire of recruitment molecules than previously expected.

Consistent with our previous findings assessing the inflammatory profile of monocyte subsets in generally healthy individuals, we found that lipid levels explained the inter-participant variations (29). Interestingly, although there was a spread in participants’ ages, there was no association between age or sex and monocyte recruitment profile. Most notably, low levels of ApoA1 were associated with higher levels of both adhesion molecules and chemokine receptors. Whether this is causal, in that ApoA1 impacts recruitment marker expression, or the reverse that marker expression alters ApoA1 levels, was not investigated. However, an increased potential to extravasate when ApoA is low is consistent with studies showing that incubating monocytes with ApoA1 can reduce monocyte diapedesis towards a range of chemokines under both acute and chronic conditions *in vitro* (26, 45). Because chemokines and chemokine receptors are critical in the development of atherosclerosis, modulation of their expression by ApoA1 suggest that low ApoA1 levels may allow increased monocyte recruitment. Though ApoA1 is a key protein associated with HDL, no associations were evident between markers examined and HDL-C. This may reflect the fact that ApoA1 mediates many of the inflammatory effects of HDL, including reduction of CD11b on monocytes (27). Some clinical studies indicate ApoA1 may be a better prognostic marker than LDL-C or HDL-C in prediction of severity of coronary artery disease (46, 47); its link with monocyte recruitment may factor into this.

The finding that the expression of CCR2 is associated with participants’ LDL-C levels is consistent with previous findings of patients with hypercholesterolemia, including familial hypercholesterolemia (48–51).

As alterations in adhesion molecule and chemokine receptors impacts monocyte migration (49), our findings here suggests that changes in monocyte recruitment may be occurring, undetected, in individuals who are considered generally healthy even though they have dyslipidemia. This is important as a high proportion of adults [two thirds in Australia (52)] have dyslipidemia, many of whom would be untreated.

****The expression of CD29 being associated with the participants’ glucose levels suggests that adhesion potential of all monocyte subsets may be affected by elevated glucose levels. Indeed, various studies have shown that the levels of glucose impact on the expression of adhesion molecules (53–55). While increased monocyte migration into plaques has been seen in diabetes, whether this is due to increased levels of recruitment markers is unclear, as there is also increased myelopoiesis in diabetes**** (56)****. Similarly, in other comorbidities, such as hypertension (HTN) increased monocyte adhesion occurs (57–59) and thus the level of expression of the markers examined here, may be increased further in established CVD and in particular in those with co-morbidities.****

Overall, the finding that most of the associations between the monocyte adhesion molecule or chemokine receptor expression were with participants’ ApoA1 levels suggest that therapies aimed to lower LDL-C or TC alone might not aid in full reversal of this acquired adhesive or migratory phenotype of monocytes, and thus therapies elevating ApoA1 levels should also be considered.

In summary, our results are consistent with the understanding that monocyte subsets differentially express adhesion molecules and chemokine receptors which would broadly dictate their recruitment potiential. However, these inter-subset differences are overshaddowed by differences between individuals, with all monocytes in some individuals having a greater potential to migrate and to do so using a wider repertoire of recruitment markers than currently appreciated. The findings in this study, combined with our previous observation that inflammatory markers, such as cytokines, are also raised on all monocytes relative to lipid levels indicates that all monocytes, not just one subset, can acquire an increased pro-atherogenic phenotype in the circulation itself i.e. even before they become macrophages. The impact of this on plaque development is yet unclear. However, monocyte adoption of pro-atherogenic phenotype would be expected to promote plaque development as they would enter the vessel wall primed to become pro-atherogenic macrophages. Together with other contributing risk factors, monocyte priming may exacerbate atherosclerosis development. That most associations were found with ApoA1 suggests that to effectively reduce monocyte migration into the plaques to slow/inhibit plaque progression, the lowering of LDL-C alone may not be sufficient.

## Data Availability Statement

The datasets presented in this article are not readily available because we have not sought consent from the participants for the release of the data.

## Ethics Statement

The studies involving human participants were reviewed and approved by the Western Sydney Local Health District (WSLHD) Human Research Ethics Committee. The participants provided their written informed consent to participate in this study.

## Author Contributions

VP designed and performed the experiments, analyzed the results, performed statistical analysis, created the figures, and wrote the manuscript. HM planned the project, and with HW supervised the work and contributed to data interpretation and manuscript writing. SL identified suitable patients and with VP collected participant information. The project was conducted under JF. All authors contributed to the article and approved the submitted version.

## Funding

This study was supported by Westmead Medical Research Foundation (WMRF) and Clinical Chemistry Research and Education Fund.

## Conflict of Interest

The authors declare that the research was conducted in the absence of any commercial or financial relationships that could be construed as a potential conflict of interest.

## References

[1] Libby P Nahrendorf M Swirski FK. Monocyte heterogeneity in cardiovascular disease. Semin Immunopathol (2013) 35:553–62. 10.1007/s00281-013-0387-3 PMC3755757 23839097

[2] Medbury HJ James V Ngo J Hitos K Wang Y Harris DC. Differing association of macrophage subsets with atherosclerotic plaque stability. Int Angiol (2013) 32:74–84. 23435395

[3] Swirski FK Pittet MJ Kircher MF Aikawa E Jaffer FA Libby P. Monocyte accumulation in mouse atherogenesis is progressive and proportional to extent of disease. Proc Natl Acad Sci U.S.A. (2006) 103:10340–5. 10.1073/pnas.0604260103 PMC1502459 16801531

[4] Combadiere C Potteaux S Rodero M Simon T Pezard A Esposito B. Combined inhibition of CCL2, CX3CR1, and CCR5 abrogates Ly6C(hi) and Ly6C(lo) monocytosis and almost abolishes atherosclerosis in hypercholesterolemic mice. Circulation (2008) 117:1649–57. 10.1161/CIRCULATIONAHA.107.745091 18347211

[5] Noels H Weber C Koenen RR. Chemokines as Therapeutic Targets in Cardiovascular Disease. Arterioscler Thromb Vasc Biol (2019) 39:583–92. 10.1161/ATVBAHA.118.312037 30760014

[6] Leon B Ardavin C. Monocyte migration to inflamed skin and lymph nodes is differentially controlled by L-selectin and PSGL-1. Blood (2008) 111:3126–30. 10.1182/blood-2007-07-100610 18184867

[7] Ley K Laudanna C Cybulsky MI Nourshargh S. Getting to the site of inflammation: the leukocyte adhesion cascade updated. Nat Rev Immunol (2007) 7:678–89. 10.1038/nri2156 17717539

[8] Imhof BA Aurrand-Lions M. Adhesion mechanisms regulating the migration of monocytes. Nat Rev Immunol (2004) 4:432–44. 10.1038/nri1375 15173832

[9] Min JK Kim YM Kim SW Kwon MC Kong YY Hwang IK. TNF-related activation-induced cytokine enhances leukocyte adhesiveness: induction of ICAM-1 and VCAM-1 via TNF receptor-associated factor and protein kinase C-dependent NF-kappaB activation in endothelial cells. J Immunol (2005) 175:531–40. 10.4049/jimmunol.175.1.531 15972689

[10] Johnson-Leger C Imhof BA. Forging the endothelium during inflammation: pushing at a half-open door? Cell Tissue Res (2003) 314:93–105. 10.1007/s00441-003-0775-4 12955495

[11] Braunersreuther V Mach F Steffens S. The specific role of chemokines in atherosclerosis. Thromb Haemost (2007) 97:714–21. 10.1160/TH07-01-0036 17479181

[12] Tacke F Alvarez D Kaplan TJ Jakubzick C Spanbroek R Llodra J. Monocyte subsets differentially employ CCR2, CCR5, and CX3CR1 to accumulate within atherosclerotic plaques. J Clin Invest (2007) 117:185–94. 10.1172/JCI28549 PMC1716202 17200718

[13] Ziegler-Heitbrock L Ancuta P Crowe L Dalod M Grau V Hart DN. Nomenclature of monocytes and dendritic cells in blood. Blood (2010) 116:e74–80. 10.1182/blood-2010-02-258558 20628149

[14] Cros J Cagnard N Woollard K Patey N Zhang SY Senechal B. Human CD14dim monocytes patrol and sense nucleic acids and viruses via TLR7 and TLR8 receptors. Immunity (2010) 33:375–86. 10.1016/j.immuni.2010.08.012 PMC3063338 20832340

[15] Boyette LB Macedo C Hadi K Elinoff BD Walters JT Ramaswami B. Phenotype, function, and differentiation potential of human monocyte subsets. PloS One (2017) 12:e0176460. 10.1371/journal.pone.0176460 28445506 PMC5406034

[16] Ancuta P Rao R Moses A Mehle A Shaw SK Luscinskas FW. Fractalkine preferentially mediates arrest and migration of CD16+ monocytes. J Exp Med (2003) 197:1701–7. 10.1084/jem.20022156 PMC2193954 12810688

[17] Thomas GD Hamers AAJ Nakao C Marcovecchio P Taylor AM Thomas GD. Human Blood Monocyte Subsets: A New Gating Strategy Defined Using Cell Surface Markers Identified by Mass Cytometry. Arterioscler Thromb Vasc Biol (2017) 37:1548–58. 10.1161/ATVBAHA.117.309145 PMC5828170 28596372

[18] Wong KL Tai JJ Wong WC Han H Sem X Yeap WH. Gene expression profiling reveals the defining features of the classical, intermediate, and nonclassical human monocyte subsets. Blood (2011) 118:e16–31. 10.1182/blood-2010-12-326355 21653326

[19] Kapellos TS Bonaguro L Gemund I Reusch N Saglam A Hinkley ER. Human Monocyte Subsets and Phenotypes in Major Chronic Inflammatory Diseases. Front Immunol (2019) 10:2035. 10.3389/fimmu.2019.02035 31543877 PMC6728754

[20] Swirski FK Libby P Aikawa E Alcaide P Luscinskas FW Weissleder R. Ly-6Chi monocytes dominate hypercholesterolemia-associated monocytosis and give rise to macrophages in atheromata. J Clin Invest (2007) 117:195–205. 10.1172/JCI29950 17200719 PMC1716211

[21] Soehnlein O Drechsler M Doring Y Lievens D Hartwig H Kemmerich K. Distinct functions of chemokine receptor axes in the atherogenic mobilization and recruitment of classical monocytes. EMBO Mol Med (2013) 5:471–81. 10.1002/emmm.201201717 PMC3598085 23417922

[22] Rogacev KS Cremers B Zawada AM Seiler S Binder N Ege P. CD14++CD16+ monocytes independently predict cardiovascular events: a cohort study of 951 patients referred for elective coronary angiography. J Am Coll Cardiol (2012) 60:1512–20. 10.1016/j.jacc.2012.07.019 22999728

[23] Stansfield BK Ingram DA. Clinical significance of monocyte heterogeneity. Clin Trans Med (2015) 4:5. 10.1186/s40169-014-0040-3 PMC4384980 25852821

[24] Rogacev KS Seiler S Zawada AM Reichart B Herath E Roth D. CD14++CD16+ monocytes and cardiovascular outcome in patients with chronic kidney disease. Eur Heart J (2011) 32:84–92. 10.1093/eurheartj/ehq371 20943670

[25] Tolani S Pagler TA Murphy AJ Bochem AE Abramowicz S Welch C. Hypercholesterolemia and reduced HDL-C promote hematopoietic stem cell proliferation and monocytosis: studies in mice and FH children. Atherosclerosis (2013) 229:79–85. 10.1016/j.atherosclerosis.2013.03.031 23684512 PMC3691284

[26] Iqbal AJ Barrett TJ Taylor L McNeill E Manmadhan A Recio C. Acute exposure to apolipoprotein A1 inhibits macrophage chemotaxis in vitro and monocyte recruitment in vivo. Elife (2016) 5. 10.7554/eLife.15190 PMC5030090 27572261

[27] Murphy AJ Woollard KJ Hoang A Mukhamedova N Stirzaker RA McCormick SP. High-density lipoprotein reduces the human monocyte inflammatory response. Arterioscler Thromb Vasc Biol (2008) 28:2071–7. 10.1161/ATVBAHA.108.168690 18617650

[28] Hyka N Dayer JM Modoux C Kohno T Edwards CK 3rd Roux-Lombard P. Apolipoprotein A-I inhibits the production of interleukin-1beta and tumor necrosis factor-alpha by blocking contact-mediated activation of monocytes by T lymphocytes. Blood (2001) 97:2381–9. 10.1182/blood.V97.8.2381 11290601

[29] Patel VK Williams H Li SCH Fletcher JP Medbury HJ. Monocyte inflammatory profile is specific for individuals and associated with altered blood lipid levels. Atherosclerosis (2017) 263:15–23. 10.1016/j.atherosclerosis.2017.05.026 28570862

[30] Nichols M Peterson K Herbert J Alston L Allender S National Heart Foundation of Australia, issuing body. Australian heart disease statistics. Physical activity and cardiovascular disease. Melbourne: National Heart Foundation of Australia (2016).

[31] Williams H Cassorla G Pertsoulis N Patel V Vicaretti M Marmash N. Human classical monocytes display unbalanced M1/M2 phenotype with increased atherosclerotic risk and presence of disease. Int Angiol J Int Union Angiol (2017) 36:145–55. 10.23736/S0392-9590.16.03661-0 26871397

[32] Kratofil RM Kubes P Deniset JF. Monocyte Conversion During Inflammation and Injury. Arteriosclerosis Thrombosis Vasc Biol (2017) 37:35–42. 10.1161/ATVBAHA.116.308198 27765768

[33] Connaughton EP Naicker S Hanley SA Slevin SM Eykelenboom JK Lowndes NF. Phenotypic and functional heterogeneity of human intermediate monocytes based on HLA-DR expression. Immunol Cell Biol (2018). 10.1111/imcb.12032 29505094

[34] Ziegler-Heitbrock L Hofer TP. Toward a refined definition of monocyte subsets. Front Immunol (2013) 4:23. 10.3389/fimmu.2013.00023 23382732 PMC3562996

[35] Wong KL Yeap WH Tai JJ Ong SM Dang TM Wong SC. The three human monocyte subsets: implications for health and disease. Immunol Res (2012) 53:41–57. 10.1007/s12026-012-8297-3 22430559

[36] Hamers AAJ Dinh HQ Thomas GD Marcovecchio P Blatchley A Nakao CS. Human Monocyte Heterogeneity as Revealed by High-Dimensional Mass Cytometry. Arteriosclerosis Thrombosis Vasc Biol (2019) 39:25–36. 10.1161/ATVBAHA.118.311022 PMC6697379 30580568

[37] Weber C Belge KU von Hundelshausen P Draude G Steppich B Mack M. Differential chemokine receptor expression and function in human monocyte subpopulations. J Leukoc Biol (2000) 67:699–704. 10.1002/jlb.67.5.699 10811011

[38] Weber C Shantsila E Hristov M Caligiuri G Guzik T Heine GH. Role and analysis of monocyte subsets in cardiovascular disease. Joint consensus document of the European Society of Cardiology (ESC) Working Groups “Atherosclerosis & Vascular Biology” and “Thrombosis”. Thromb Haemost (2016) 116:626–37. 10.1160/TH16-02-0091 27412877

[39] Askenase MH Han SJ Byrd AL Morais da Fonseca D Bouladoux N Wilhelm C. Bone-Marrow-Resident NK Cells Prime Monocytes for Regulatory Function during Infection. Immunity (2015) 42:1130–42. 10.1016/j.immuni.2015.05.011 PMC4472558 26070484

[40] Quintin J Saeed S Martens JH Giamarellos-Bourboulis EJ Ifrim DC Logie C. Candida albicans infection affords protection against reinfection via functional reprogramming of monocytes. Cell Host Microbe (2012) 12:223–32. 10.1016/j.chom.2012.06.006 PMC3864037 22901542

[41] Shalova IN Lim JY Chittezhath M Zinkernagel AS Beasley F Hernandez-Jimenez E. Human monocytes undergo functional re-programming during sepsis mediated by hypoxia-inducible factor-1alpha. Immunity (2015) 42:484–98. 10.1016/j.immuni.2015.02.001 25746953

[42] Mandl M Schmitz S Weber C Hristov M. Characterization of the CD14++CD16+ monocyte population in human bone marrow. PloS One (2014) 9:e112140. 10.1371/journal.pone.0112140 25369328 PMC4219836

[43] Rogacev KS Zawada AM Hundsdorfer J Achenbach M Held G Fliser D. Immunosuppression and monocyte subsets. Nephrol Dialysis Transplant: Off Publ Eur Dialysis Transplant Assoc - Eur Renal Assoc (2015) 30:143–53. 10.1093/ndt/gfu315 25313167

[44] Guo N Chen Y Su B Yang X Zhang Q Song T. Alterations of CCR2 and CX3CR1 on Three Monocyte Subsets During HIV-1/Treponema pallidum Coinfection. Front Med (Lausanne) (2020) 7:272. 10.3389/fmed.2020.00272 32626718 PMC7314900

[45] Diederich W Orso E Drobnik W Schmitz G Apolipoprotein AI. and HDL(3) inhibit spreading of primary human monocytes through a mechanism that involves cholesterol depletion and regulation of CDC42. Atherosclerosis (2001) 159:313–24. 10.1016/S0021-9150(01)00518-4 11730811

[46] Florvall G Basu S Larsson A. Apolipoprotein A1 is a stronger prognostic marker than are HDL and LDL cholesterol for cardiovascular disease and mortality in elderly men. J Gerontol Ser A Biol Sci Med Sci (2006) 61:1262–6. 10.1093/gerona/61.12.1262 17234819

[47] Garfagnini A Devoto G Rosselli P Boggiano P Venturini M. Relationship between HDL-cholesterol and apolipoprotein A1 and the severity of coronary artery disease. Eur Heart J (1995) 16:465–70. 10.1093/oxfordjournals.eurheartj.a060937 7671890

[48] Han KH Han KO Green SR Quehenberger O. Expression of the monocyte chemoattractant protein-1 receptor CCR2 is increased in hypercholesterolemia. Differential effects of plasma lipoproteins on monocyte function. J Lipid Res (1999) 40:1053–63. 10.1016/S0022-2275(20)33509-4 10357837

[49] Bernelot Moens SJ Neele AE Kroon J van der Valk FM Van den Bossche J Hoeksema MA. PCSK9 monoclonal antibodies reverse the pro-inflammatory profile of monocytes in familial hypercholesterolaemia. Eur Heart J (2017) 38:1584–93. 10.1093/eurheartj/ehx002 28329114

[50] Verweij SL Duivenvoorden R Stiekema LCA Nurmohamed NS van der Valk FM Versloot M. CCR2 expression on circulating monocytes is associated with arterial wall inflammation assessed by 18F-FDG PET/CT in patients at risk for cardiovascular disease. Cardiovasc Res (2018) 114:468–75. 10.1093/cvr/cvx224 29186373

[51] Han KH Tangirala RK Green SR Quehenberger O. Chemokine receptor CCR2 expression and monocyte chemoattractant protein-1-mediated chemotaxis in human monocytes. A regulatory role for plasma LDL. Arteriosclerosis Thrombosis Vasc Biol (1998) 18:1983–91. 10.1161/01.ATV.18.12.1983 9848893

[52] Nichols M PK Herbert J Alston L Allender S. Australian heart disease statistics 2015 (2016). Melbourne: National Heart Foundation of Australia (Accessed March 2017).

[53] Peschel T Niebauer J. Role of pro-atherogenic adhesion molecules and inflammatory cytokines in patients with coronary artery disease and diabetes mellitus type 2. Cytometry B Clin Cytom (2003) 53:78–85. 10.1002/cyto.b.10026 12717696

[54] Bouma G Lam-Tse WK Wierenga-Wolf AF Drexhage HA Versnel MA. Increased serum levels of MRP-8/14 in type 1 diabetes induce an increased expression of CD11b and an enhanced adhesion of circulating monocytes to fibronectin. Diabetes (2004) 53:1979–86. 10.2337/diabetes.53.8.1979 15277376

[55] Ceriello A Falleti E Motz E Taboga C Tonutti L Ezsol Z. Hyperglycemia-induced circulating ICAM-1 increase in diabetes mellitus: the possible role of oxidative stress. Horm Metab Res (1998) 30:146–9. 10.1055/s-2007-978854 9566857

[56] Nagareddy PR Murphy AJ Stirzaker RA Hu Y Yu S Miller RG. Hyperglycemia promotes myelopoiesis and impairs the resolution of atherosclerosis. Cell Metab (2013) 17:695–708. 10.1016/j.cmet.2013.04.001 23663738 PMC3992275

[57] Tummala PE Chen XL Sundell CL Laursen JB Hammes CP Alexander RW. Angiotensin II induces vascular cell adhesion molecule-1 expression in rat vasculature: A potential link between the renin-angiotensin system and atherosclerosis. Circulation (1999) 100:1223–9. 10.1161/01.CIR.100.11.1223 10484544

[58] Shalia KK Mashru MR Vasvani JB Mokal RA Mithbawkar SM Thakur PK. Circulating levels of cell adhesion molecules in hypertension. Indian J Clin Biochem (2009) 24:388–97. 10.1007/s12291-009-0070-6 PMC3453057 23105866

[59] Tropea BI Huie P Cooke JP Tsao PS Sibley RK Zarins CK. Hypertension-enhanced monocyte adhesion in experimental atherosclerosis. J Vasc Surg (1996) 23:596–605. 10.1016/S0741-5214(96)80038-3 8627894

